# The Response Microbial of the Cucumber Rhizosphere Network Keystone Taxa of the Cucumber Rhizosphere to Continuous Fertilization

**DOI:** 10.3390/microorganisms13020451

**Published:** 2025-02-18

**Authors:** Jiaquan Tang, Haoyang Wu, Yaqian Li, Jie Chen

**Affiliations:** 1 School of Agriculture and Biology, Shanghai Jiao Tong University, Shanghai 200240, China; tangjiaquan@sjtu.edu.cn (J.T.); gwfihy_atopos@sjtu.edu.cn (H.W.); yaqianli2008@163.com (Y.L.); 2 The State Key Laboratory of Microbial Metabolism, Shanghai Jiao Tong University, Shanghai 200240, China

**Keywords:** continuous fertilization, microbial network, rhizosphere microbial community, structural equation model, keystone taxa

## Abstract

Fertilization is a common agricultural practice used to modify the physicochemical properties of soil, which in turn affects plant growth and the rhizosphere microbial community. However, the mechanisms underlying the variation in the cucumber rhizosphere microecosystem have not been thoroughly investigated. In this study, we conducted three rounds of continuous plant growth experiments in pots to test different fertilizers and reveal the evolutionary features of the rhizosphere microecosystem. Through topological analysis of the microbial co-occurrence networks, we identified putative taxa associated with fertilization disturbances. Structural equation models (SEMs) predict plausible mechanistic links between soil physicochemical properties, plant growth and the rhizosphere microbiome. The results suggest that continuous fertilization with single fertilizers reduces microbial diversity and may disrupt the structure of the microbial network. Furthermore, it was found that the predicted distribution of keystone taxa (*Bacteroidetes*, *Ascomycota*, etc.) was significantly sensitive to the application of certain fertilizers. Moreover, it was modeled by the SEMs that the accumulation of NO_3_^−^ and Na^+^ in fertilized soil was one of the putative principal causes of rhizosphere microbial network deterioration. This study provides new insights into the dynamic changes in the cucumber rhizosphere microbial community under continuous fertilization and highlights the potential utility of SEMs in analyzing causal relationships in agroecosystem studies before experimental validation.

## 1. Introduction

Plant rhizosphere microbes are essential to maintaining soil nutrients and structure, as well as plant growth and protection [1,2]. These microorganisms make the soil a living system through a series of biochemical reactions. Rhizosphere microbiomes are crucial for plant growth and health, and these microbes are also referred to as having the secondary genome of plants [3]. The importance of the soil microbiota and the functions of certain taxa have been comprehensively studied [4]. Recently, research on the effects of soil physicochemical property changes on soil microbiota compositions has increased in recent years. However, network dynamics and keystone taxa of the crop rhizosphere microbiota in continuously fertilized monocropping agroecosystems have rarely been studied.

Cucumbers are widely consumed vegetables that contribute significantly to human health. They are rich in vitamins, particularly vitamin C, minerals, and antioxidants, which promote hydration, support immune function, and provide antioxidant benefits [5]. Cucumbers are low in calories, making them a popular choice for maintaining a healthy diet. In addition to their nutritional value, cucumbers also possess anti-inflammatory and anti-cancer properties, highlighting their importance in human health beyond basic nutrition.

Continuous fertilization in agricultural practices plays a crucial role in enhancing microbial diversity within rhizosphere soil. Fertilizers supply essential nutrients that support the growth and development of soil microorganisms, leading to a more diverse microbial community. Studies have shown that proper fertilization practices can increase both microbial richness and functional diversity in the rhizosphere, which is vital for nutrient cycling, disease suppression, and soil structure maintenance [6,7]. Moreover, continuous fertilization influences the abundance of specific microbial taxa that contribute positively to plant health and growth.

Microbial diversity in the rhizosphere of crops, such as cucumbers, is essential for promoting plant growth and health. The microorganisms in the rhizosphere are involved in critical processes such as nitrogen fixation, organic matter decomposition, and pathogen suppression, which enhance nutrient availability, disease resistance, and overall plant growth [8]. For example, beneficial bacteria and fungi in the rhizosphere facilitate nutrient uptake and produce metabolites that inhibit harmful pathogens. The diversity of these microorganisms is a key factor in optimizing soil health and improving crop productivity.

In recent years, microbiota network topological analysis has been increasingly applied in various soil ecological studies to investigate interactions between microbial taxa and predict the structure of microbial communities across temporal or spatial gradients [9]. Researchers identified keystone bacterial genera in soil samples and models predict that phyla such as *Proteobacteria* and *Actinobacteria* played a key role as connectors in the microbial network [10]. The relationship between agricultural intensification and the microbial network suggests a strong negative correlation between agricultural intensification and root fungal network connectivity [11]. Previous studies primarily focused on the alpha and beta diversity of soil microbiota. However, in contrast, network analysis delves deeper into the co-occurrence patterns among soil microorganisms. This approach not only uncovers potential biotic interactions but also reveals shared physiologies, which can guide more comprehensive investigations into microbial community dynamics [12].

One of the most pragmatic applications of microbial network analysis is to identify the potential keystone taxa in each microbial community [10]. Studies on the dynamics of microbial communities revealed that keystone taxa may be indicators of community shifts [13]. In addition, spatiotemporal heterogeneity has been used to study the abundance and distribution of keystone taxa in soil [14]. To date, studies on keystone taxa have primarily focused on the identification and potential of keystone taxa in different ecosystems. We hypothesize that disturbances in agroecosystems significantly alter the composition and function of keystone taxa, yet the specific effects on these taxa remain poorly understood.

In the study, microbial networks of cucumber rhizosphere soil treated with different fertilizers were constructed to study the disturbance of fertilization on rhizosphere microbial networks and keystone taxa. We aimed (1) that the sensitivity of rhizosphere microbial network to different types of fertilizers should be different, and the structure of predicted microbial network might be reshaped by various fertilizers, (2) that some predicted keystone taxa might be more sensitive to disturbance of fertilization than the other keystone taxa and more adaptive to various fertilizers treatments, (3) that fertilizer might affect the rhizosphere microbiota directly through changing soil physicochemical properties and indirectly through affecting the growth of cucumber. Keystone taxa of microbial networks with different fertilizer treatments were identified and compared to reveal their variation under different fertilizer treatments. Considering the soil, plant, and rhizosphere microbiota as the interacting components of the rhizosphere microecosystem, we constructed SEMs to model putative direct and indirect causal effects among the components of the cucumber rhizosphere microecosystem.

## 2. Materials and Methods

### 2.1. Experimental Design

Three cycles (30 d per cycle) of pot-based (14 cm × 17 cm, diameter × height) experiments were conducted in a greenhouse (25 °C) at Shanghai Jiaotong University. Each pot, containing 2.5 kg of homogenized no-tillage soil, was planted with two cucumber seedlings (*Cucumis sativus* cv. Shenqing 4, catalog number: SN-SQ4-2022; lot: L20220915; Shanghai Shengnong Seed Co., Ltd., Shanghai, China). The cucumber seeds (Cucumis sativus cv. Shenqing 4) were commercially procured from Shanghai Shengnong Seed Co., Ltd. (Shanghai, China). Prior to planting, the seeds were surface sterilized with 2% sodium hypochlorite (NaClO) for 5 min, rinsed thoroughly with sterile water, and germinated on moist filter paper at 28 °C in the dark for 48 h. The potting soil (soil type, Eutric Gleysols; FAO classification, Ge62-2/3a) was collected from the topsoil layer (0–20 cm depth) of a no-tillage agricultural field in a suburban area of Shanghai, China (31°00′40.4″ N, 121°23′45.6″ E). After collection, the soil underwent standardized pre-treatment: visible plant residues, stones, and roots were manually removed. The soil was air-dried under ambient laboratory conditions for 7 days, passed through a 2-mm mesh sieve to ensure particle size uniformity, and thoroughly homogenized to minimize spatial heterogeneity. No sterilization or chemical treatment was applied to preserve the natural soil microbiota. Cucumber seedlings were raised on a peat-based substrate (70% sphagnum peat, 20% vermiculite, 10% perlite; pH 6.2 ± 0.3; Shangcheng Horticultural Supplies Co., Ltd., Shanghai, China) before pot transplantation. Fertilization treatments were designed as follows: (1) CK, control group without fertilization; (2) IA, acidic NPK chemical fertilizer: ammonium sulfate (1.906 g/pot; Catalog No.: AS-2021-05; Lot: L20220512; Manufacturer: Sinochem Group, Beijing, China), diammonium phosphate (0.362 g/pot; Catalog No.: DAP-2021-08; Lot: L20220822; Manufacturer: Yara International, Oslo, Norway), potassium chloride (0.799 g/pot; Catalog No.: KCl-2022-03; Lot: L20220315; Manufacturer: K+S Group, Kassel, Germany); (3) IB, alkaline NPK chemical fertilizer: ammonium bicarbonate (2.284 g/pot; Catalog No.: ABC-2021-10; Lot: L20221018; Manufacturer: BASF SE, Ludwigshafen, Germany), diammonium phosphate (0.362 g/pot; Catalog No.: DAP-2021-08; Lot: L20220822; Manufacturer: Yara International, Oslo, Norway), potassium chloride (0.799 g/pot; Catalog No.: KCl-2022-03; Lot: L20220315; Manufacturer: K+S Group, Kassel, Germany); (4) IN, neutral NPK chemical fertilizer: urea (0.865 g/pot; Catalog No.: U-2022-01; Lot: L20220120; Manufacturer: CF Industries, Northbrook, IL, USA), diammonium phosphate (0.362 g/pot; Catalog No.: DAP-2021-08; Lot: L20220822; Manufacturer: Yara International, Oslo, Norway), potassium chloride (0.799 g/pot; Catalog No.: KCl-2022-03; Lot: L20220315; Manufacturer: K+S Group, Kassel, Germany); (5) OF, bioorganic fertilizer Wangtaibao (Dajing Bio-tech, Shanghai, China): *Trichoderma harzianum* wettable powder (23.1 g/pot; Catalog No.: TH-WP-2022; Lot: L20221105; Manufacturer: Dajing Bio-Tech Co., Ltd., Shanghai, China), http://www.dajingbio.com/productinfo/583004.html (accessed on 15 October 2024); (6) CF, compound fertilizer Nitrophoska (EuroChem Group AG, Zug, Switzerland): Nitrophoska (1.155 g/pot; Catalog No.: NPK-15-15-15-2021; Lot: L20211201; Manufacturer: EuroChem Group AG, Zug, Switzerland), N:P_2_O_5_:K_2_O = 15%:15%:15%, https://eurochemagro.com/products/nitrophoska/ (accessed on 16 October 2024). The doses of three types of NPK chemical fertilizers were designed according to a previous report about cucumber fertilization [15]. Bioorganic fertilizer and compound fertilizer were applied according to the product instruction manuals of Wangtaibao (Wangtaibao^®^ Trichoderma harzianum wettable powder, Dajing Bio-Tech Co., Ltd., Shanghai, China) and Nitrophoska (Nitrophoska^®^ NPK 15-15-15, EuroChem Group AG, Zug, Switzerland) were applied according to their respective product instruction manuals.

Each treatment contained four replicates and was arranged in a random block design. All pots were equally watered with 200 mL of water every two days to maintain soil moisture at approximately 70% of field capacity. The plants were grown under a 12/12 h light-dark photoperiod at a temperature of 25 ± 2 °C and a relative humidity of 60 ± 5%. At the end of each cycle, cucumber plants were harvested, and new cucumber seedlings were replanted immediately after collecting the soil and plant samples [16]. We harvested the cucumbers upon maturity and then continued to sow cucumber seeds in the pots until the third harvest. Samples collected at third cropping rounds were used for further research in this study.

### 2.2. Sample Collection

After harvesting, plant root weight (RW) and fresh weight (FW) were measured immediately. Rhizosphere soil samples were collected by gently shaking the roots to remove loosely attached soil, followed by brushing off the soil tightly adhering to the roots. The collected soil samples were immediately stored at −80 °C for subsequent analysis of the rhizosphere microbiome, as described by Wang et al. [17]. Soil samples in each pot treated with different fertilizers were collected with an auger (2 cm × 10 cm, diameter × depth) and divided into three samples for soil mineral N analysis, soil water content (WC) and chemical analysis.

### 2.3. Evaluation of Soil Physicochemical Properties and Plant Health

Soil mineral N, including NO_3_^−^ and NH_4_^+^, was measured according to the method of Caires et al. [18] with a Smartchem 170 (AMSAlliance, Frépillon, France). Soil samples were dried at 105 °C for 12 h to measure the soil WC. The following physicochemical properties were analyzed with air-dried soil samples: pH, determined with a glass electrode (soil/water = 1/2.5); electrical conductivity (EC), measured with an EC meter (soil/water = 1/5); soluble cations (Na^+^, K^+^, Ca^2+^, and Mg^2+^), determined with an atomic absorption spectrometer (PerkinElmer, Shelton, CT, USA). The cucumber disease index (DI) was measured according to the method of Chen et al. [19] at the end of each cycle.

### 2.4. Rhizosphere Soil DNA Extraction and NovaSeq Sequencing

A heterogenized no-tillage soil sample was referred to as the background soil sample. Rhizosphere soil samples were collected by harvesting cucumber roots. Soil directly adhering to cucumber roots was collected and pooled together from four plants grown in four different pots, as described earlier [20]. Total genomic DNA was extracted from soil samples using a FastDNA SPIN Kit (MP Biomedicals, Shanghai, China) and quantified using a Nanodrop 2000 spectrophotometer (Thermo Scientific, Waltham, MA, USA). The DNA samples were amplified with the primers 341F (5′-ACTCCTACGGGAGGCAGCAG-3′) and 805R (5′-GACTACHVGGGTATCTAATCC-3′), which target the bacterial 16S rRNA V3-V4 region. The primers ITS1 (5′-CTTGGTCATTTAGAGGAAGTAA-3′) and ITS2 (5′-GCTGC-GTTCTTCATCGATGC-3′) were used to amplify the fungal internal transcribed spacer ITS1 region. Each sample was amplified in triplicate in 50 μL reactions, and the following thermal cycling conditions were applied: initial denaturation at 95 °C for 2 min; 35 cycles at 94 °C for 20 s, 55 °C for 40 s and 72 °C for 60 s; final extension at 72 °C for 10 min. The quality of the PCR products was analyzed with 2% agarose gel electrophoresis, and the products were purified with an Agarose Gel DNA purification kit (Axygen, Union City, CA, USA). Then, purified PCR products were quantified by a Quant-iT PicoGreen dsDNA Assay kit (Thermo Fisher Scientific, USA) and a microplate reader (BioTek, FLx800, Shoreline, WA, USA). Next, the samples were mixed according to the sequencing requirements of each sample. A sequencing library for each sample was then prepared with the TruSeq Nano DNA LT Library Prep kit (Illumina, Shanghai, China). Finally, the sequencing library of each sample was sequenced with an Illumina NovaSeq sequencer and a NovaSeq 6000 SP Reagent Kit (500 cycles) (Illumina, Shanghai, China). Sequence data were deposited in the NCBI database with the accession numbers PRJNA612659 (for bacteria) and PRJNA612665 (for fungi).

### 2.5. NovaSeq Sequencing Data Processing

Raw sequencing data were processed using the QIIME2 pipeline (version 2021.4) http://qiime2.org/ (accessed on 19 August 2024). Initially, raw data were denoised and clustered using DADA2 in QIIME2 to obtain amplicon sequence variants (ASVs) [21]. ASVs were annotated with the classify-sklearn algorithm in QIIME2 [22] with reference to the Silva (Release132) http://www.arb-silva.de (accessed on 25 August 2024) and UNITE (Release 8.0) https://unite.ut.ee/ (accessed on 3 September 2024) databases for bacteria and fungi, respectively. The Chao1 index and Pielou’s evenness were calculated using QIIME2 to describe the microbial community richness and evenness of each sample [23,24]. A Bray–Curtis-based clustering tree was constructed based on the unweighted pair-group method with arithmetic means (UPGMA) algorithm and viewed using R (v3.5.2) to review the similarity between microbial community compositions of different treatments.

### 2.6. Network Analysis

To study the co-occurrence patterns among the different groups of rhizosphere bacterial and fungal communities, network analysis was conducted using NovaSeq sequence data [6]. Co-occurrence networks were constructed and visualized using ‘igraph’ and ‘ggraph’ packages in R (v3.5.2). Topological measures for constructed networks (vertex number and degree of centralization) were calculated according to the method of Csardi et al. [25]. Topological indices (Zi, within-module connectivity; Pi, among-module connectivity) for a single node in the network were also calculated to identify the keystone taxa of the microbial community and the nodes with Pi > 0.62 or Zi > 2.5 were identified as the keystone taxa in the microbial network [26].

### 2.7. Statistical Analysis

Analysis of variance (ANOVA) for the biotic and abiotic factors was calculated using R (v3.5.2). Redundancy analysis (RDA) for microbial keystone taxa was performed using the ‘vegan’ package and visualized using the ‘ggplot2’ package of R (v3.5.2). A heatmap of Spearman’s rank correlations was calculated and visualized using the ‘psych’ and ‘corrplot’ packages of R (v3.5.2). SEMs were constructed based on the results of Spearman’s rank correlations of the biotic and abiotic factors of the rhizosphere microecosystem, and the goodness of fit of SEMs was evaluated with the ‘lavaan’ package of R (v3.5.2). The visualization of SEMs was realized using the ‘semplot’ package of R (v3.5.2).

## 3. Results

### 3.1. Soil Properties and Cucumber Growth

**Figure 1:**
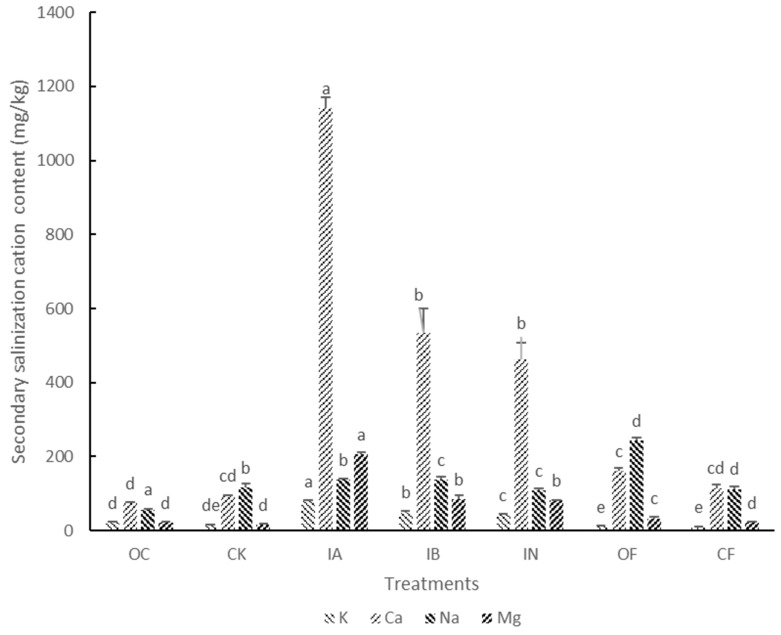
Content of secondary salinization cations in different fertilizer treatments. Values with the same letter are not significantly different (*p* < 0.05). K, Soil soluble K^+^ content; Ca, Soil soluble Ca^2+^ content; Na, Soil soluble Na^+^ content; Mg, Soil soluble Mg^2+^ content.

Basic datasets, including soil physicochemical properties and results of cucumber growth, are summarized in Table 1 and Figure 1. Monocropping of cucumber without any fertilizer (CK) significantly increased the soil pH. Nevertheless, acidification of the soil was observed in the three NPK chemical fertilizer-treated samples (IA, IB, and IN) regardless of the pH of the fertilizers. Meanwhile, no significant acidification was found in compound fertilizer (CF) and bioorganic (OF) fertilizer-treated soils. The contents of the soil soluble Ca^2+^, Mg^2+^, K^+^, Na^+^, and NO_3_^−^ were significantly increased in the three NPK fertilizer treatments, and the application of NPK chemical fertilizers with acidic pH resulted in the highest level of secondary salinization cations. In addition, the application of urea resulted in the highest level of NO_3_^−^ after three cycles of continuous fertilization. Compared with the control treatment (CK), the application of NPK and compound fertilizers (IA, IB, IN, and CF) suppressed the growth of cucumber roots. Moreover, groups treated with the application of NPK and compound fertilizers had higher disease indexes than the control group, while bioorganic fertilizer reduced the disease occurrence rate of cucumber.

The soil ASV richness (BC and FC) and microbial community composition evenness (BP and FP) of the soil microbiota values are summarized in Table 2. The results showed that the monocropping of cucumber significantly reduced the bacterial OTU community richness and evenness throughout the monocropping process (OC and CK). In addition, the application of chemical NPK (IA and IN) and compound fertilizer (CF) led to lower bacterial OTU richness and evenness compared with the group without fertilization. The use of bioorganic fertilizer also reduced bacterial OTU richness after three rounds of intensive application. As for the variation of rhizosphere fungi, the application of all kinds of fertilizers significantly decreased the rhizosphere soil fungal OTU richness compared with that of the background (OC) soil microbiota throughout the monocropping process.

### 3.2. Changes in Rhizosphere Microbial Network Structure Caused by Continuous Fertilization

A topological analysis of the microbiome network was conducted to determine putative variations of the rhizosphere microbiota ecological structure under continuous fertilization. Network scale and connectivity are shown in Table 3. The growth of cucumber without fertilization (CK) significantly increased the predicted bacterial network scale and connectivity, while the application of neutral NPK fertilizer (IN), compound fertilizer (CF) and bioorganic fertilizer (OF) significantly reduced the network size. In addition, all kinds of fertilizers led to a lower bacterial network connectivity compared with the control group (CK). Among all the fertilizers, the compound fertilizer resulted in the lowest bacterial network scale and connectivity. In addition, bioorganic fertilizer and compound fertilizer were observed to reduce the fungal network size and connectivity under intensive application for three cycles.

### 3.3. Predicted Keystone Taxa Related to Fertilizer Treatments

**Figure 2:**
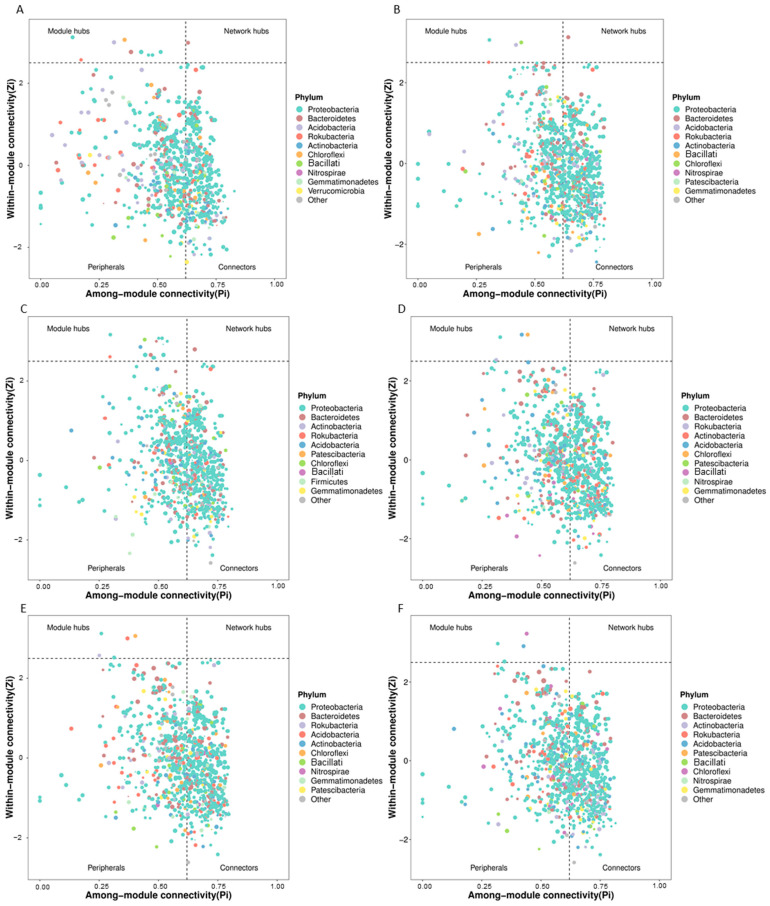
Predicted keystone rhizosphere bacterial taxa of soil samples under the application of different types of fertilizer in a monocropping agroecosystem. With the topological parameters Zi and Pi of each taxon in the bacterial microbiota network, the taxa of all networks were divided into four types: peripherals, connectors, module hubs, and network hubs. A network hub is taxa with high connectivity for the whole network. Module hubs represent the taxa with high connectivity in a module. Connectors are the taxa with high connectivity between two modules. Peripheral taxa do not have high connectivity within or between modules. Generally, the three types of taxa other than peripherals are classified as keystone taxa. (**A**): Control group. (**B**): Bioorganic fertilizer treatment. (**C**): Compound fertilizer treatment. (**D**): Acidic NPK chemical fertilizer treatment. (**E**): Alkaline NPK chemical fertilizer treatment. (**F**): Neutral NPK chemical fertilizer treatment.

In addition to the study of microbial network variations, keystone taxa of each fertilizer treatment were also predicted. As shown in Figure 2, the keystone bacterial taxa of the control group (Figure 2A) were the phyla Proteobacteria, Bacteroidetes, Acidobacteria, Rokubacteria, Actinobacteria, Chloroflexi, Bacillati, Nitrospirae, Gemmatimonadetes, and Verrucomicrobia. Furthermore, taxa from Proteobacteria, Bacteroidetes, Chloroflexi, Rokubacteria, and Acidobacteria were arranged as the module hubs of the microbial network. Additionally, a node from Bacteroidetes was classified as the network hub. Compared with the control treatment, the application of bioorganic fertilizer resulted in a reduced number of module hubs, and taxa from Bacteroidetes and Rokubacteria model networks were no longer the module hub in this network model (Figure 2B). In contrast, the application of compound fertilizer increased the number of module hubs of *Proteobacteria* (Figure 2C). Moreover, the application of NPK chemical fertilizer led to a decline in module hubs and network hub *Bacteroidetes* disappeared in the three chemical fertilizer treated groups (Figure 2D–F). Precisely, the module hubs in soil treated with NPK fertilizers at acidic and neutral pH levels were Proteobacteria, Acidobacteria, and Chloroflexi. Four module hubs found in alkaline NPK chemical fertilizer-treated soil were Proteobacteria, Acidobacteria, Chloroflexi, and Nitrospirae.

**Figure 3:**
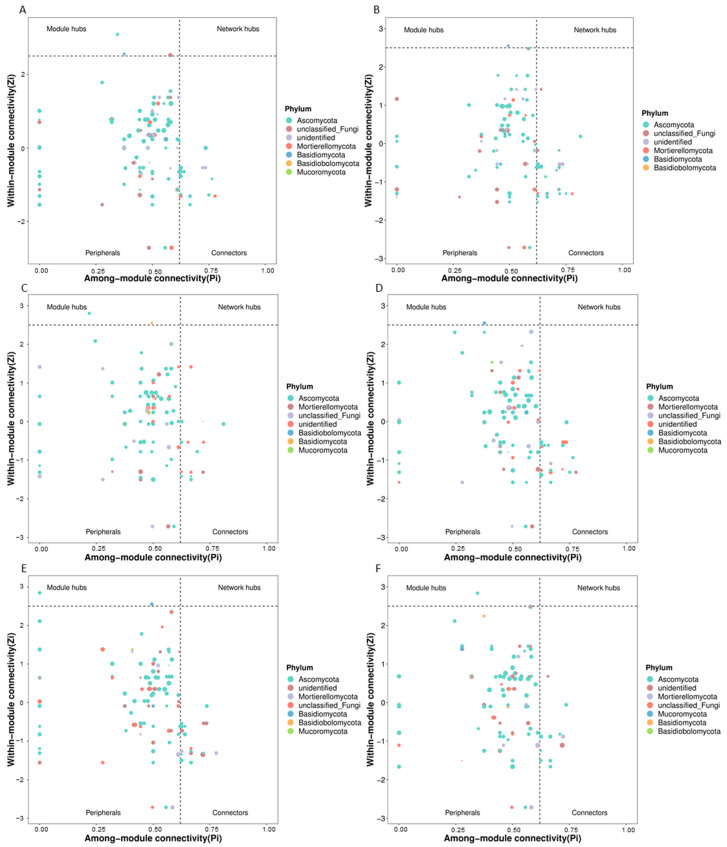
Keystone rhizosphere fungal taxa of soil samples under the application of different types of fertilizer in a monocropping agroecosystem. With the topological parameters Zi and Pi of each taxon in the fungal microbiota network, the taxa of every network were divided into four types: peripherals, connectors, module hubs, and network hubs. The network hub is taxa with high connectivity for the whole network. The module hub represents the taxa with high connectivity in a module. The connector is the taxa with high connectivity between two modules. Peripheral taxa do not have high connectivity within or between modules. Generally, the three types of taxa other than peripherals are classified as keystone taxa. (**A**): Control group. (**B**): Bioorganic fertilizer treatment. (**C**): Compound fertilizer treatment. (**D**): Acidic chemical NPK fertilizer treatment. (**E**): Basic chemical NPK chemical fertilizer treatment. (**F**): Neutral chemical fertilizer treatment.

The predicted fungal keystone taxa of the different treatments are shown in Figure 3. Ascomycota, Basidiomycota, and Mortierellomycota, as well as a kind of unidentified fungus, were detected as the fungal keystone taxa in the control group (Figure 3A). Two taxa, one from Ascomycota and another from Basidiomycota, were identified as module hubs in the control group, while no fungal network hub was found in any treatment. Additionally, compared with the control group, Ascomycota was no longer the module hub with the application of bioorganic fertilizer (Figure 3B) and NPK chemical fertilizer with acidic pH (Figure 3D). Moreover, Basidiomycota was no longer a member of a module hub in samples that had been treated with the neutral chemical NPK fertilizer (Figure 3F).

### 3.4. Structural Equation Model Construction

**Figure 4:**
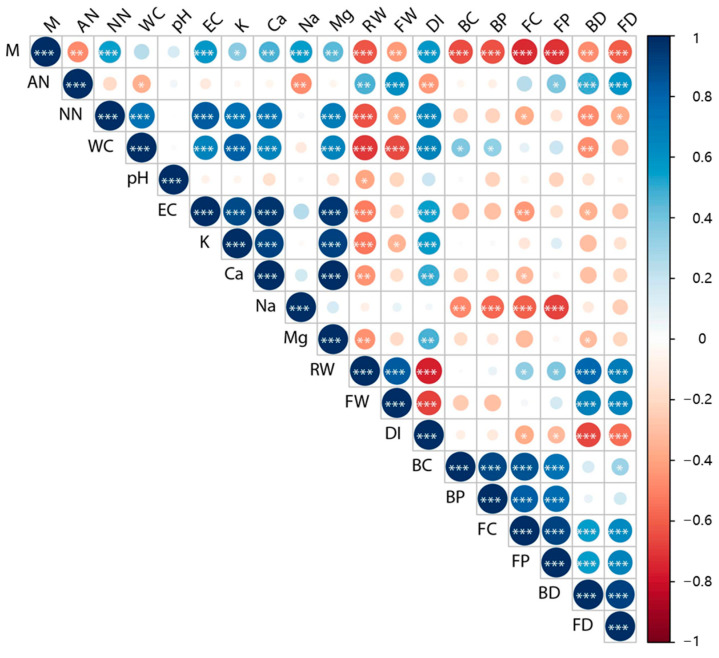
Heatmap of Spearman’s rank correlations between the soil physicochemical properties, plant growth results and the plant rhizosphere microbiota properties. The correlation coefficients are indicated by color intensity, and the significance levels are marked with asterisks (*: *p* < 0.05, **: *p* < 0.01, ***: *p* < 0.001). Abbreviations are as follows: WC, soil water content; pH, soil pH; EC, soil conductivity; AN, soil ammonia nitrogen content; NN, soil nitrate nitrogen content; K, soil soluble K^+^ content; Ca, soil soluble Ca^2+^ content; Na, soil soluble Na^+^ content; Mg, soil soluble Mg^2+^ content; RW, cucumber root fresh weight; DI, plant disease index; BC, bacterial Chao1 index; BP, bacterial Pielou’s evenness; BD, bacterial network degree centralization; FC, fungal Chao1 index; FP, fungal Pielou’s evenness; FV, fungal network vertex number; FD, fungal network degree centralization.

To understand the influence of continuous fertilization on plant growth and the rhizosphere microecosystem of cucumber, a correlation analysis was conducted on the soil physicochemical factors, plant growth, and rhizosphere microbial properties (Figure 4). The accumulation of salinization factors, including soil-soluble K^+^, Ca^2+^, Mg^2+^, and NO^3−^, significantly suppressed root growth (RW) of cucumber (*p* < 0.01). In addition, the soil water content (WC) was positively correlated with the bacterial abundance (BC) and evenness (BP) (*p* < 0.05). The accumulation of soluble Na+ in soil was negatively correlated with soil bacterial and fungal community richness and evenness (*p* < 0.01). The plant RW was positively (*p* < 0.001) correlated with microbial network scale and connectivity (BD and FD), while the plant DI was negatively correlated with microbial network connectivity (*p* < 0.001). Additionally, soil BD and FD were negatively correlated with soil NO^3−^ content (*p* < 0.05).

**Figure 5:**
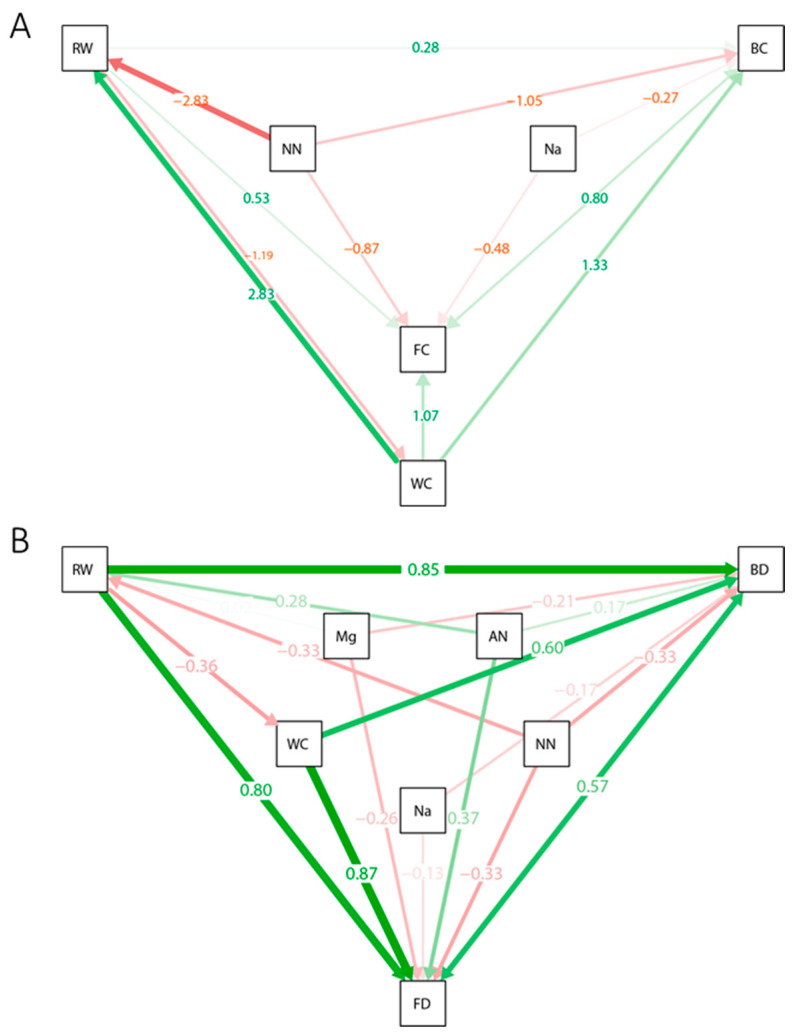
Structural equation models (SEMs) illustrating the direct and indirect effects of soil physicochemical properties and plant growth on the rhizosphere microbiome community abundances (**A**) and the microbial network connectivity (**B**). Solid arrows represent significant relationships (*p* < 0.05). The arrow color indicates the nature of the correlation (red, negative; green, positive), and the thickness of the arrow indicates the strength of the relationship. Standardized path coefficients are shown on the pathways. The goodness of fit for the models is as follows: A, χ2 = 1.961, Df = 2, *p*-value = 0.375, GFI = 0.998, CFI = 1, RMSEA = 0; B, χ2 = 0.008, Df = 1, *p*-value = 0.929, GFI = 1, CFI = 1, and RMSEA = 0. The meanings of abbreviations are the same as those in Figure 4.

Based on a correlation analysis of the soil–fertilizer–plant ecosystem, SEMs were then constructed to model the putative mechanistic variation in the rhizosphere microecosystem under continuous fertilization practices (Figure 5). The results of the causal model suggested that high levels of soil Na^+^ and NO^3−^ are the main salinization factors that decrease rhizosphere microbiome richness (BC and FC) (Figure 5A). In addition, high levels of soil NO^3−^ declined the diversity of the rhizosphere microbiota community indirectly by suppressing the growth of cucumber (Figure 5A). When it comes to the result of OTU microbial network connectivity, apart from soil Na+ and NO^3−^, the accumulation of soil Mg^2+^ also resulted in a decline in microbial network connectivity (Figure 5B). Moreover, an indirect suppressive effect of NO^3−^ accumulation on the microbial network connectivity through plant growth was also found in the causal model (Figure 5B). In addition, ammonia nitrogen (AN) was found to positively influence the growth of plant roots and the connectivity of the rhizosphere network.

## 4. Discussion

### 4.1. Intensive Fertilization Degraded the Rhizosphere

Agricultural intensification is one of the most serious problems that led to the deterioration of soil biotic and abiotic properties in various agroecosystems during the 21st century [27]. Previous studies have focused on the results of soil degradation [28], while the evolutionary process of the rhizosphere microecosystem has been less studied. In this study, we observed that soil acidification and secondary salinization were induced by NPK chemical fertilizers through continuous fertilization (Table 1 and Figure 1), which was in line with results from previous research [29,30]. In addition, no significant acidification and accumulation of secondary salinization factors were found in compound fertilizer and organic fertilizer-treated soils, which indicated that these two types of fertilizers maintain better soil physicochemical properties [31].

A previous study revealed that continuous application of chemical fertilization decreased the abundance of the soil microbial community [16]. In this study, we observed that the application of acidic and neutral NPK fertilizers and compound fertilizers significantly reduced the evenness and richness of the cucumber rhizosphere microbiota (Table 2). It was reported that bioorganic fertilizer maintained a more stable soil microbial community compared with chemical fertilizer, but the effect of the intensive application of a single bioorganic fertilizer on the rhizosphere microbiome has rarely been studied [32]. Our research revealed that with the continuous application of bioorganic fertilizer, cucumber rhizosphere microbial abundance decreased after three rounds of fertilization. This phenomenon might be due to the excessive rate of fertilizer application [33].

### 4.2. Rhizosphere Microbial Networks Were Reshaped by Continuous Fertilization

Previous studies have mainly focused on the richness and diversity of the rhizosphere microbiota [34]. However, in the present study, we investigated the variation of predicted microbial networks during the continuous application of different fertilizers. Compared with the background soil, rhizosphere predicted microbial network size and connectivity were observed to increase significantly with the cropping of cucumber. This might be due to an increase in nutrition secreted by cucumber roots, as the nutrition secreted by plant roots played an important role in shaping the rhizosphere microbial network [35]. Additionally, continuous fertilization with various fertilizers intensified the deterioration of the rhizosphere microbial network connectivity in continuous fertilized treatments (Table 3). Moreover, the continuous application of compound fertilizer resulted in the lowest rhizosphere bacterial community richness and evenness (CF), which might be due to the low soil WC caused by the use of compound fertilizer according to SEM results (Figure 5B) [36]. Moreover, continuous application of neutral NPK chemical fertilizer (IN) led to a lower network size and connectivity compared with NPK chemical fertilizers with acidic and alkaline pH, which suggests that cucumber rhizosphere microbial community network structure may be more sensitive to neutral NPK chemical fertilizer.

### 4.3. Response of Rhizosphere Microbial Keystone Taxa to Fertilizers

Keystone taxa, as indicators of a microbial network, are expected to be essential for the stability, function and evolution of a microbial community [14]. In this study, we predicted and compared the compositions of rhizosphere microbial network keystone taxa under different fertilizer treatments (Figure 2 and Figure 3). The phylum Bacteroidetes was identified as the rhizosphere network hub of the control group (Figure 2A). A previous study revealed that Bacteroidetes was a sensitive bioindicator for agricultural soil usage [37]. In this study, we found that Bacteroidetes was more sensitive to NPK chemical fertilizers than the other types of fertilizers as it was no longer the network hub of the predicted microbial community for three NPK chemical fertilizers treated soils (Figure 2D–F). Research about the response of soil Proteobacteria to various environmental stresses indicated that this phylum exhibited a complex lifestyle, making it adaptable to different environmental disturbances [38]. Our research supported this finding by revealing that Proteobacteria was expected to be the module hubs for all types of fertilizer treatments, which indicated that this phylum was resistant to the disturbance of continuous fertilization.

Compared with the rhizosphere bacterial network, the predicted fungal network was smaller and simpler (Table 3), and a fungal network hub was not found in any of the treatments in this research (Figure 3). The fungal phylum Ascomycota was previously identified as a keystone fungal taxon for the rhizosphere microbial network of the genus Buxus [39]. Similarly, in the present study, Ascomycota was identified as a module hub of the cucumber rhizosphere microbial network. However, the application of bioorganic fertilizer and acidic NPK chemical fertilizer removed Ascomycota as a member of the network hubs, which suggests that Ascomycota may be sensitive to the continuous application of bioorganic fertilizer and acidic NPK fertilizer (Figure 3B,D). Additionally, the application of neutral NPK fertilizer excluded Basidiomycota as a member of module hubs (Figure 3F), which indicated that the keystone taxon Basidiomycota may be sensitive to the application of neutral NPK chemical fertilizer [40].

### 4.4. Rhizosphere Microbial Network Variation Unveiled by Structural Equation Models

The multivariate method was the main statistical method used to identify the causal factors of soil ecological variation in recent decades [41]. To date, first-generation multivariate methods, including canonical correspondence analysis (CCA), principal components analysis (PCA), and nonmetric multidimensional scaling (NMS), have been widely used to reveal patterns in soil ecosystem analysis [42]. However, these methods could not model the networks of causal relationships and the indirect effects among the studied ecological variables, thus limiting the mechanistic understanding of soil ecology. To predict plausible the mechanistic links between soil physicochemical properties, plant growth, and the rhizosphere microbial community, we proposed two multivariate hypotheses (Figure 5) based on the results of correlation analysis of the variables (Figure 4). A second-generation multivariate method, SEM, was then applied to support these hypothetical network models [43].

Fertilization influences the rhizosphere microbial community by regulating the soil properties and plant growth [44]. The results of SEMs (Figure 5) predict the accumulation of the soil salinization factors Na^+^ and NO^3−^ by continuous fertilization as the main cause of the decrease in rhizosphere microbial richness [45]. In addition, the soil NO^3−^ content also predicts a decrease in microbial richness by suppressing the growth of plants (Figure 5A). This result suggests the indirect effect of NO^3−^ on the rhizosphere microbiome [46] (Figure 5A). The application of urea (INF and INT) resulted in the highest accumulation of NO^3−^ among all the three NPK chemical fertilizer treatments (Table 1). Accordingly, the microbial richness of the urea-treated soil was also lower than that of the other NPK fertilizer-treated groups (Table 2). In this case, we proposed that soil NO^3−^ accumulation could be used as an indicator of the effect of continuous NPK chemical fertilizer application. The remediation of NPK fertilizer-degraded soil should be focused on reducing the use of urea and removing excessive soil NO^3−^ [47]. Moreover, soil NO^3−^ also significantly suppressed the formation of the rhizosphere microbial network (Figure 5B), which highlights the importance of NO^3−^ in the rhizosphere microecosystem. Apart from the accumulation of soil NO^3−^, other secondary salinization factors, including soil Na^+^ and Mg^2+^ should also be considered to protect the rhizosphere microbial network (Figure 5B). Increased plant root growth was found to be favourable for microbial community richness and network complexity, and the plant can also reduce the content of soil NO^3−^ which was harmful to the rhizosphere microbial community; thus, we propose growing salt-tolerant plants on the overfertilized soil and reduce the frequency of fertilizer application to restore the microbial network of degraded soil (Figure 4 and Figure 5) [48].

### 4.5. Limitations and Future Directions

While our structural equation models (SEMs) and network analyses provide valuable insights into the rhizosphere microecosystem dynamics, these approaches are inherently correlative. The co-occurrence patterns and keystone taxa identified here reflect statistical associations rather than experimentally confirmed interactions. To transition from correlation to causation, future work should integrate: (1) Gnotobiotic experiments to test the functional roles of keystone taxa (e.g., Bacteroidetes and Ascomycota) under controlled conditions. (2) Targeted manipulation of soil NO^−3^ and Na^+^ levels to validate their causal effects on microbial network deterioration. (3) Metatranscriptomic or metabolomic analyses to elucidate molecular mechanisms underlying observed patterns.

## 5. Conclusions

Our research revealed that the continuous application of a single fertilizer developed the situation of serious soil degradation and thereby suppressed the rhizosphere microbial community. Some types of fertilizers, such as NPK chemical fertilizers with acidic and alkaline pH, sustained the microbial network structure better compared with the other fertilizers in this research. Predicted keystone taxa sensitive to different types of fertilizer were identified in this study, and it reveals that bacterial phyla *Bacteroidetes* as a network hub may be sensitive to the three types of NPK chemical fertilizers. The fungal phyla *Ascomycota* was identified as a module hub and may be sensitive to bioorganic fertilizer and NPK chemical fertilizer with acidic pH. These identified fertilization-sensitive keystone taxa could be used as an indicator for the fertilized soil microbial network quality. Finally, the construction of SEM models provided a network model to illustrate the plausible variation mechanism of the rhizosphere micro-ecosystem, and it predicts the causal effects among soil physicochemical properties, plant growth and soil microbial community. In conclusion, our research provided a reference theoretical model for the fertilization practice for the maintenance and remediation of the soil ecosystem.

## Figures and Tables

**Table 1:** Soil physicochemical properties and plant growth.

Sample	Water Content (%)	pH	EC (μS cm^−1^)	NH_4_^+^-N (mg kg^−1^)	NO_3_^−^-N (mg kg^−1^)	Root Weight (g Plant^−1^)	Fresh Weight (g Plant^−1^)	Disease Index
OC	30.86 ± 2.72 ^c^	7.22 ± 0.02 ^d^	111.33 ± 0.77 ^f^	6.72 ± 0.79 ^ab^	89.81 ± 4.31 ^c^	NA	NA	NA
CK	23.8 ± 1.46 ^d^	7.81 ± 0.05 ^a^	186.33 ± 14.83 ^e^	5.61 ± 0.18 ^cd^	37.58 ± 6.21 ^c^	0.52 ± 0.07 ^ab^	6.04 ± 0.43 ^e^	1.83 ± 0.35 ^e^
IA	38.51 ± 6.94 ^a^	6.82 ± 0.03 ^f^	1149.67 ± 15.63 ^a^	7.07 ± 1.08 ^ab^	571.82 ± 77.46 ^b^	0.33 ± 0.08 ^d^	12.96 ± 2.54 ^c^	2.67 ± 0.44 ^b^
IB	40.88 ± 3.43 ^a^	7.03 ± 0.01 ^e^	689.33 ± 69.81 ^c^	6.47 ± 0.46 ^bc^	563.74 ± 92.89 ^b^	0.22 ± 0.06 ^e^	7.42 ± 1.25 ^de^	3.83 ± 0.35 ^a^
IN	35.63 ± 2.07 ^b^	6.99 ± 0.03 ^e^	759 ± 10 ^b^	7.21 ± 0.57 ^ab^	879.07 ± 59.13 ^a^	0.27 ± 0.05 ^e^	12.55 ± 0.98 ^c^	2.67 ± 0.69 ^b^
OF	18.8 ± 1.1 ^e^	7.44 ± 0.01 ^c^	377.67 ± 14.73 ^d^	5.55 ± 0.71 ^d^	40.4 ± 2.52 ^c^	0.61 ± 0.17 ^a^	18.68 ± 2.96 ^a^	1.5 ± 0.46 ^f^
CF	14.6 ± 0.48 ^f^	7.54 ± 0.03 ^b^	182.87 ± 12.4 ^e^	7.27 ± 1.49 ^a^	59.73 ± 8.51 ^c^	0.4 ± 0.06 ^c^	14.93 ± 2.58 ^b^	2.5 ± 0.46 ^c^

Values represent the mean ± standard error (*n* = 3). Values with the same lowercase letter are not significantly different (*p* < 0.05). Data in a column followed by a different lowercase letter indicate a significant difference at the 0.05 probability level based on protected LSD tests. NA: Not applicable.

**Table 2:** Estimated observed richness (Chao1 index) and evenness (Pielou’s evenness) for the different soil samples.

Sample	BC	BP	Shannon–Wiener (H’)	FC	FP	Shannon–Wiener (H’)
OC	7519.33 ^a^	0.887762 ^a^	6.45 ^a^	545.747 ^a^	0.696502 ^a^	4.12 ^a^
CK	5114.23 ^c^	0.769883 ^b^	5.89 ^b^	508.907 ^b^	0.601616 ^b^	3.78 ^b^
IA	4304.61 ^d^	0.726326 ^c^	5.45 ^c^	292.24 ^d^	0.579451 ^c^	3.45 ^c^
IB	5271.89 ^b^	0.700113 ^d^	5.67 ^d^	380.1 ^c^	0.488957 ^e^	3.12 ^d^
IN	3699.93 ^e^	0.655445 ^e^	5.23 ^e^	259.066 ^e^	0.496514 ^d^	2.98 ^e^
OF	2979.23 ^f^	0.615297 ^f^	4.89 ^f^	123.904 ^g^	0.16346 ^g^	2.45 ^f^
CF	2215.64 ^g^	0.598955 ^g^	4.56 ^g^	134.658 ^f^	0.21207 ^f^	2.23 ^g^

Values with the same lowercase letter are not significantly different (*p* < 0.05). Data in a column followed by a different lowercase letter indicate a significant difference at the 0.05 probability level based on protected LSD tests. BC Bacterial Chao1 index. BP Bacterial Pielou’s evenness. FC Fungal Chao1 index. FP Fungal Pielou’s evenness.

**Table 3:** Topology indices of the soil microbiome network.

Sample	Bacterial		Fungal		Shannon–Wiener (H’)
	Vertex Number	Degree Centralization	Vertex Number	Degree Centralization	
OC	352 ^d^	15,784 ^e^	81 ^c^	804 ^de^	3.45 ^b^
CK	726 ^a^	116,520 ^a^	80 ^c^	1626 ^ab^	4.12 ^a^
OF	612 ^b^	56,234 ^bc^	52 ^d^	896 ^d^	3.78 ^c^
CF	501 ^c^	39,354 ^d^	50 ^d^	742 ^e^	3.56 ^d^
IA	682 ^a^	57,138 ^bc^	83 ^c^	1531 ^bc^	4.05 ^a^
IB	711 ^a^	63,670 ^b^	80 ^c^	1682 ^a^	4.10 ^a^
IN	612 ^b^	49,478 ^c^	77 ^c^	1494 ^c^	3.98 ^b^

Vertex number: Number of nodes in the studied microbiome network; a higher vertex number indicates a larger microbial network. Degree centralization: Network degree centralization is calculated based on the degree of all nodes in the microbiome network; a higher degree centralization indicates that a network contains more connections between nodes. Shannon–Wiener (H’) index: A measure of microbial diversity, with higher values indicating greater diversity. Values with the same lowercase letter are not significantly different (*p* < 0.05).

## Data Availability

The original contributions presented in the study are included in the article; further inquiries can be directed to the corresponding author.

## References

[1] Wang Y., Li T., Li Y., Björn L.O., Rosendahl S. (2021). Long-term fertilization alters the complexity and stability of soil microbial networks in a wheat-maize cropping system. Soil Biol. Biochem..

[2] Domeignoz-Horta L.A., Cappelli S.L., Shrestha R., Gerin S., Lohila A.K., Heinonsalo J., Nelson D.B., Kahmen A., Duan P., Sebag D. (2024). Plant diversity drives positive microbial associations in the rhizosphere enhancing carbon use efficiency in agricultural soils. Nat. Commun..

[3] Zhang X., Li H., Liu Y., Wang Z., Zhang Y., Chen S. (2023). Rhizosphere microbiomes as a secondary genome: A novel perspective on plant growth and health. Nat. Microbiol..

[4] Wang L., Zhang L., Yang Q., Liu H., Chen L., Zhang M., Guo J. (2024). Soil microbiota diversity and the functional roles of key taxa in soil health and plant growth. Environ. Microbiol..

[5] Kumar A., Singh R., Yadav A., Yadav P. (2024). Cucumber (*Cucumis sativus* L.) Phytochemicals and Their Health Promoting Benefits: A Comprehensive Review. J. Food Sci. Technol..

[6] Zhang L., Yuan L., Ai C., Zhang H., Zhou W. (2024). Maize functional requirements drive the selection of rhizobacteria under long-term fertilization practices. New Phytol..

[7] Li X., Liu X., Zhang Z., Zhao Y., Chen L., Yang J., Xu Y., He Y. (2019). Effects of fertilization on the rhizosphere microbial community and plant growth in a cucumber cultivation system. Appl. Soil Ecol..

[8] Zhang Y., Zhang X., Wei Z., Yang H., Shen Q., Zhang R. (2021). Rhizosphere microorganisms as key drivers of nutrient cycling and disease suppression in agroecosystems. Environ. Microbiol. Rep..

[9] Mouquet N., Dossantos M., Thébault E., Guillemette T., Loreau M. (2019). Ecological network analysis reveals the impact of biodiversity loss on ecosystem functioning. Nat. Commun..

[10] Banerjee S., Kirkby C.A., Schmutter D., Bissett A., Kirkegaard J.A., Richardson A.E. (2016). Network analysis reveals functional redundancy and keystone taxa amongst bacterial and fungal communities during organic matter decomposition in an arable soil. Soil Biol. Biochem..

[11] Gao L., Yang L., Xu J., Li X., Wang S., Zhang R. (2021). Microbial network analysis reveals the negative effect of agricultural practices on fungal community structure and connectivity in soil. Appl. Soil Ecol..

[12] Yuan H., Ge T., Zhou P., Liu S., Roberts P., Zhu H., Zou Z., Tong C., Wu J. (2013). Soil microbial biomass and bacterial and fungal community structures responses to long-term fertilization in paddy soils. J. Soils Sediments.

[13] Wang J., Zhang Y., Zhang W., Li L., Xu S., Liu Z., Guo S. (2020). Spatiotemporal variation in the abundance and distribution of keystone taxa in soil: Implications for soil health and ecosystem services. Soil Biol. Biochem..

[14] Herren C.M., McMahon K.D. (2018). Keystone taxa predict compositional change in microbial communities. Environ. Microbiol..

[15] Huang S., Tang J., Li C., Zhang H., Yuan S. (2017). Reducing potential of chemical fertilizers and scientific fertilization countermeasure in vegetable production in China. J. Plant Nutr. Fertil..

[16] Cai F., Pang G., Li R.-X., Li R., Gu X.-L., Shen Q.-R., Chen W. (2017). Bioorganic fertilizer maintains a more stable soil microbiome than chemical fertilizer for monocropping. Biol. Fertil. Soils.

[17] Wang G., Xu Y., Jin J., Liu J., Zhang Q., Liu X. (2009). Effect of soil type and soybean genotype on fungal community in soybean rhizosphere during reproductive growth stages. Plant Soil.

[18] Caires E.F., Filho R.Z., Barth G., Joris H.A. (2016). Optimizing nitrogen use efficiency for no-till corn production by improving root growth and capturing NO_3_-N in subsoil. Pedosphere.

[19] Chen L., Yang X., Raza W., Li J., Liu Y., Qiu M., Zhang F., Shen Q. (2011). Trichoderma harzianum SQR-T037 rapidly degrades allelochemicals in rhizospheres of continuously cropped cucumbers. Appl. Microbiol. Biotechnol..

[20] Zhang F., Zhu Z., Yang X., Ran W., Shen Q. (2013). Trichoderma harzianum T-E5 significantly affects cucumber root exudates and fungal community in the cucumber rhizosphere. Appl. Soil Ecol..

[21] Callahan B.J., Mcmurdie P.J., Rosen M.J., Han A.W., Johnson A.J.A., Holmes S.P. (2016). DADA2: High-resolution sample inference from Illumina amplicon data. Nat. Methods.

[22] Bokulich N.A., Kaehler B.D., Rideout J.R., Dillon M., Bolyen E., Knight R., Huttley G.A., Gregory Caporaso J. (2018). Optimizing taxonomic classification of marker-gene amplicon sequences with QIIME 2’s q2-feature-classifier plugin. Microbiome.

[23] Chao A. (1984). Nonparametric estimation of the number of classes in a population. Scand. J. Stat..

[24] Pielou E.C. (1966). The measurement of diversity in different types of biological collections. J. Theor. Biol..

[25] Csardi G., Nepusz T. (2006). The igraph software package for complex network research. Int. J. Complex Syst..

[26] Deng Y., Jiang Y.-H., Yang Y., He Z., Luo F., Zhou J. (2012). Molecular ecological network analyses. BMC Bioinform..

[27] Tscharntke T., Clough Y., Wanger T.C., Jackson L., Motzke I., Perfecto I., Vandermeer J., Whitbread A. (2012). Global food security, biodiversity conservation and the future of agricultural intensification. Biol. Conserv..

[28] Wang S., Jiang H., Guo X., Yang W., Liu J., Zhang T., Huang X. (2021). Soil degradation and its impact on soil microbial communities: A review of recent research. Geoderma.

[29] Beiying Z., Tianlin C., Bing W. (2010). Effects of longterm uses of chemical fertilizers on soil quality. Chin. Agric. Sci. Bull..

[30] Zhao X., Zhang W., Liu Y., Zhang J., Chen L. (2019). Effects of long-term fertilization on soil acidification and salinization in agroecosystems: A review. Sci. Total Environ..

[31] Chen L., Liu Z., Wu Y., Sun H., Zhang Y., Zhou D. (2021). Organic and compound fertilizers improve soil properties and mitigate secondary salinization in arid regions. Agric. Ecosyst. Environ..

[32] Qiu M., Zhang R., Xue C., Zhang S., Li S., Zhang N., Shen Q. (2012). Application of bio-organic fertilizer can control *Fusarium wilt* of cucumber plants by regulating microbial community of rhizosphere soil. Biol. Fertil. Soils.

[33] Chen Z., Zhang F., He Y., Liu S., Chen Z., Xu Q. (2021). Continuous application of bioorganic fertilizer alters microbial community structure and decreases microbial abundance in cucumber rhizosphere soil. J. Soils Sediments.

[34] Liu L., Chen X., Zhang Q., Wang C., Yang J., Liu S. (2021). Shifts in the rhizosphere microbiome of tomato in response to soil types and cultivation practices. Microorganisms.

[35] Sasse J., Martinoia E., Northen T. (2018). Feed your friends: Do plant exudates shape the root microbiome?. Trends Plant Sci..

[36] de Menezes A.B., Müller C., Clipson N., Doyle E. (2016). The soil microbiome at the Gi-FACE experiment responds to a moisture gradient but not to CO_2_ enrichment. Microbiology.

[37] Li P., Wang W., Li J., Zhang H., Zhao Q. (2020). Bacteroidetes as a sensitive bioindicator of agricultural soil quality: The influence of long-term fertilization on soil microbiome structure. Soil Biol. Biochem..

[38] Bouskill N.J., Barker-Finkel J., Galloway T.S., Handy R.D., Ford T.E. (2010). Temporal bacterial diversity associated with metal-contaminated river sediments. Ecotoxicology.

[39] LeBlanc N., Crouch J.A. (2019). Prokaryotic taxa play keystone roles in the soil microbiome associated with woody perennial plants in the genus Buxus. Ecol. Evol..

[40] Sun X., Zhang Y., Ma X., Li X., Wang Y. (2020). Impact of long-term NPK fertilization on fungal communities in agricultural soils: Focusing on the exclusion of Basidiomycota. Soil Biol. Biochem..

[41] Oksanen J. (2011). Multivariate analysis of ecological communities in R: Vegan tutorial. R Package Version.

[42] Kent M. (2006). Numerical classification and ordination methods in biogeography. Prog. Phys. Geogr..

[43] Eisenhauer N., Bowker M.A., Grace J.B., Powell J.R. (2015). From patterns to causal understanding: Structural equation modeling (SEM) in soil ecology. Pedobiologia.

[44] Zhu S., Vivanco J.M., Manter D.K. (2016). Nitrogen fertilizer rate affects root exudation, the rhizosphere microbiome and nitrogen-use-efficiency of maize. Appl. Soil Ecol..

[45] Wang J., Zhang H., Li X., Gao L., Zhang Y. (2018). Effects of continuous fertilization on soil salinity and microbial community in a rice-wheat rotation system. Environ. Sci. Pollut. Res..

[46] Hamlin R.L., Barker A.V. (2006). Influence of ammonium and nitrate nutrition on plant growth and zinc accumulation by Indian mustard. J. Plant Nutr..

[47] Mosier A., Doran J., Freney J. (2002). Managing soil denitrification. J. Soil Water Conserv..

[48] Liu C., Yang X., Liu Y., Zhang X., Li L. (2019). Root exudates regulate the rhizosphere microbiome in a maize–wheat intercropping system. ISME J..

